# Protein Meta-Functional Signatures from Combining Sequence, Structure, Evolution, and Amino Acid Property Information

**DOI:** 10.1371/journal.pcbi.1000181

**Published:** 2008-09-26

**Authors:** Kai Wang, Jeremy A. Horst, Gong Cheng, David C. Nickle, Ram Samudrala

**Affiliations:** 1 Computational Genomics Group, Department of Microbiology, University of Washington, Seattle, Washington, United States of America; 2 Center for Applied Genomics, Children's Hospital of Philadelphia, Philadelphia, Pennsylvania, United States of America; 3 Department of Oral Biology, University of Washington, Seattle, Washington, United States of America; 4 Department of Biochemistry, University of Washington, Seattle, Washington, United States of America; Stanford University, United States of America

## Abstract

Protein function is mediated by different amino acid residues, both their positions and types, in a protein sequence. Some amino acids are responsible for the stability or overall shape of the protein, playing an indirect role in protein function. Others play a functionally important role as part of active or binding sites of the protein. For a given protein sequence, the residues and their degree of functional importance can be thought of as a signature representing the function of the protein. We have developed a combination of knowledge- and biophysics-based function prediction approaches to elucidate the relationships between the structural and the functional roles of individual residues and positions. Such a meta-functional signature (MFS), which is a collection of continuous values representing the functional significance of each residue in a protein, may be used to study proteins of known function in greater detail and to aid in experimental characterization of proteins of unknown function. We demonstrate the superior performance of MFS in predicting protein functional sites and also present four real-world examples to apply MFS in a wide range of settings to elucidate protein sequence–structure–function relationships. Our results indicate that the MFS approach, which can combine multiple sources of information and also give biological interpretation to each component, greatly facilitates the understanding and characterization of protein function.

## Author Summary

Proteins are the main building blocks and functional molecules of the cell. Function is mediated by specific amino acid residues in a protein sequence, in a manner dependent on both their positions and types. Proteins are traditionally described as a sequence of amino acids and, when known, the experimentally determined coordinates of this covalently linked chain. Here we propose to expand the description of a protein to include a quantitative measure of the functional importance for each constituent amino acid. The resulting signature for a protein sequence or structure is referred to as its meta-functional signature (MFS). We present an ensemble of knowledge- and biophysics-based methods, which exploit different types of evidence for functional importance, as an automated publicly available tool to build such an MFS. We use two benchmark datasets to show that MFS can be used to identify functionally important residues from protein structure or sequence alone. Finally, we assess four diverse real-world biological questions to demonstrate the ability of MFS to give insight into the structural and functional roles of individual residues and positions, by exploiting protein sequence–structure–function relationships.

## Introduction

Vast amounts of sequence and structural data are being generated by high-throughput technologies. Functional annotations of the uncharacterized sequences and structures are significantly lagging. The time and cost of experimental techniques required to probe the function of all uncharacterized proteins are prohibitive. Therefore, computational means have been increasingly useful and popular in predicting and annotating functions for the huge amount of sequence and structure data [1],[2].

However, protein function prediction is itself a difficult problem to formulate, since it is difficult to define function [2],[3]. Various functional definition schemes (such as the Enzyme Commission [4], the Gene Ontology [5], and the SCOP superfamily [6]) have been developed over the years and have addressed various aspects of protein function. Instead of adopting an existing functional definition scheme, we proposed to probe the role of individual amino acid residues in protein function, regardless of the functional definition schemes that are used. In such cases, the protein function can be represented simply as a series of quantitative values, each of which indicates the functional importance of the corresponding amino acid residue in the protein sequence or structure. To calculate the quantitative values for each residue, we used a combined approach, the meta-functional signature (MFS), which takes into account the individual scores from various function prediction algorithms and generates a composite score for each amino acid residue in a given protein. Currently our signature generation protocol consists of the following four types of scores for four different types of information: (1) sequence conservation, (2) evolutionary conservation, (3) structural stability, and (4) amino acid type. All these scores are generated via conceptually simple and easily implementable algorithms (described below), and their combined use outperforms sophisticated algorithms that use only one source of information.

Sequence conservation is one of the most utilized methods for measuring the functional importance of individual amino acids. Amino acid residues with more conservative variation patterns are usually more important for the preservation of protein function. This concept is often used to identify the functional regions of proteins by building multiple alignments between the target sequence and all its sequence homologues, and then analyzing the degree of sequence conservation among each alignment site. Various measures of sequence conservation have been proposed over the years, with differing complexity and sophistication [7]. The simplest measures of sequence conservation are the entropy score and its variants [8]–[13]. More complicated measures [14]–[16] incorporate other information, such as amino acid pairwise similarity, physicochemical properties, and theoretical sequence profiles, into the scoring schemes. The AL2CO program package incorporates nine different scoring schemes, but these scores tend to correlate with each other [17]. Recently it was also shown that a Jensen-Shannon divergence measure improves predicting functionally important residues, and that considering conservation in sequentially neighboring sites further improves accuracy [18]. We previously demonstrated that a relative entropy measure which incorporates amino acid background frequencies, can better predict functional sites than simple entropy measures [19]. Furthermore, we found that incorporating the amino acid frequencies as estimated by the hidden Markov Models (HMMs) further improves the performance of the relative entropy measure [19]. In the current study, we use a sequence conservation measure derived from HMMs (HMM_rel_ent) as one component of our meta-functional signature generation protocol.

In addition to sequence conservation, we also incorporate evolutionary conservation information in the meta-functional signature. Many studies have shown that the use of phylogenetic relationships among a group of evolutionarily related sequences help accurate prediction of functional sites. The Evolutionary Trace method, one of the first and the most successful of such methods, analyzes residue variation patterns within and between protein subfamilies from multiple alignments, maps important residues to protein structure, and quantitatively ranks residue importance [20],[21]. A further development of the Evolutionary Trace method allows quantitative ranking of residue importance, by combining the use of evolutionary information and the entropy measures [22],[23]. Similarly, the ConSurf method constructs phylogenetic relationships from a group of similar sequences, calculates the conservation score by a Bayesian or a maximum likelihood method, and maps the conservation information to the protein surface [24],[25]. Further, a study by Soyer et al. used site-specific evolutionary models that assumed a different substitution matrix for each site, for detecting protein functional sites [26]. La et al. used evolutionary relationships among sequence fragments (phylogenetic motifs) to infer protein functional sites [27]. del Sol Mesa et al. presented several automated methods that divide a given protein family into subfamilies and search for residues that determine specificity [28]. The commonality among all these methods is that sequence relationships are analyzed based on the topology of an evolutionary tree, thus providing an additional level of information instead of relying on multiple sequence alignments alone. Here, we propose a novel method, called the state to step ratio score (SSR), for measuring evolutionary conservation. Based on given multiple alignments, we construct a maximum parsimony tree, and analyze the variation patterns from the root of the tree (theoretical ancestral sequence) to the leaf of the tree (sequences in multiple alignments) to create a score for each amino acid residue. The SSR score is a simple yet effective way of measuring evolutionary conservation.

Functional signature scores can also be derived from biophysics-based methods, using experimentally determined or computationally predicted protein structures. For example, a recent study demonstrated that destabilizing regions in protein structures can often be used to provide valuable information for functional inference and functional site identification [29]. For a given structure and a given position, we propose that we can mutate the wild-type residue to 19 other amino acids and calculate their structural stability scores, which can in turn be used to assign a score to each residue in a protein. Hence, these scores can also serve as a component of protein function prediction. We previously developed a residue-specific all-atom probability discriminatory function (RAPDF) [30] that compiles statistics from a database of experimental structures to score and pick “decoy” structures that are more likely to be similar to experimentally derived structures. The RAPDF has been optimized and enhanced in recent years for protein structure prediction [31]–[33]. Here, we further expanded the RAPDF to score residue mutations on a per-residue basis. Each residue in a given protein was mutated to one of the 19 alternative amino acids, producing new structures that were further optimized for topology (via side chain rearrangement) and maximized for stability (via global conformation perturbation). In our current MFS generation protocol, we used two RAPDF based scoring functions (RAPDF_spread and RAPDF_dif), to measure how all mutated structures deviate from each other and how the experimentally determined structure differs from mutated structures, which represent the potential impact on stability for the position and for the naturally occurring residue, respectively. These scores separate residues conserved for structure versus function.

An additional component of the meta-functional signature is information on the type of amino acids, such as histidine and cysteine, which are more likely to be located in functional sites than other amino acids. However, such “prior probability” for a functional site is not explicitly modeled and incorporated by most current functional site prediction algorithms. In our MFS generation protocol, we used 19 binary variables (all except Alanine) to represent the amino acid identity for each position in a given protein. We also examined whether the explicit use of amino acid information (for example, AAType), as opposed to the implicit use (for example, via relative entropy calculation), could provide additional information and better performance.

Given the complexity of defining and identifying protein functional sites, clearly no single method will always work to capture all protein functional site information. Therefore, several groups have begun to incorporate information from various sources, especially structure-derived information, to give more accurate predictions. Work by Chelliah et al. has shown that distinguishing the structural and functional constraints for amino acid residues leads to better prediction of protein interaction sites [34]. We have shown that by considering both structural and functional constraints on protein evolution, we can better identify functional sites and signatures [35],[36]. Recently, Petrova et al. showed that integration of seven selected sequence and structure features into a support vector machine (SVM) framework can improve identification of catalytic sites [37]. Furthermore, Fischer et al. integrated sequence conservation, amino acid distribution, predicted secondary structure and relative solvent accessibility into a probability density framework, and showed that at 20% sensitivity the integrated method leads to a 10% increase in precision over non-integrated methods for predicting catalytic residues from the Catalytic Site Atlas and PDB SITE records [38]. Youn et al. investigated the various features for discriminating catalytic from noncatalytic residues in novel structural folds, and showed that a measure of sequence conservation, a measure of structural conservation, a degree of uniqueness of a residue's structural environment, solvent accessibility, and residue hydrophobicity are the best predictors of catalytic sites [39]. Other similar studies also incorporated dozens to hundreds of features into a machine-learning framework for catalytic site identification [40],[41]. Altogether, the previous work suggests great value in using several complementary sequence and structure components for scoring catalytic sites. Unlike these approaches that were largely based on machine-learning algorithms, in the current study, we aim to combine several sources of information regarding the sequence, structure, evolution, and type of amino acids together via a simple logistic regression model for function prediction, including both catalytic sites and binding sites. The major advantage of the regression model is that each component can be associated with a biologically meaningful interpretation, and that individual scores for a protein can be manually studied to gain additional insights into different aspects of protein function, which are not available when many components are thrown into a sophisticated machine-learning framework. We compare the MFS approach with several other functional site prediction algorithms, propose enhancements to our approach, exemplify the wide definition of function assessed by MFS, and discuss how different components of MFS can be used to understand biological function via four real-world examples.

## Methods

### Components of the Meta-Functional Signatures

#### Sequence conservation score

We searched each query sequence against the Uniref90 database [42] using three iterations of the PSI-BLAST program [43] and built multiple alignments. We then compiled a HMM model using the HMMER package [44] and calculated the positional relative entropy using amino acid frequencies estimated by the HMM model.

The HMM_rel_ent score was calculated as
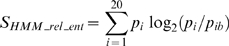
where *p_i_* (*i* = 1,…, 20) represents the amino acid emission frequency estimated by the HMM model, and *p_ib_* represents the amino acid background frequency given in the karlin.c of the BLAST program package [43].

#### Evolutionary conservation score

Using the multiple alignments generated in the above step, we built phylogenetic trees with maximum parsimony methods using the protpars program in the PHYLIP program package [45]. When several equally parsimonious trees existed, we used the first tree. For each aligned position, we then calculate the state to step ratio (SSR) as
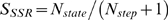
where *N_state_* is the number of residue types at a given alignment position and *N_step_* is the total number of residue type changes in the position as inferred from the root of the tree.

#### Structural stability score

We used a residue-specific all-atom probability discriminatory function (RAPDF) score as an indicator of structural stability. The RAPDF score is based on the conditional probability of a conformation being native-like, given a set of inter-atomic distances. The detailed formulation of the RAPDF score is described elsewhere [30],[31]. The original version of this function was used as a key component of our protein structure prediction methods that work well in the CASP blind prediction experiments [33],[46]. In the current study, we used a modified version of the RAPDF score [32], the 37-bin RAPDF, by using distance bins of 0.5Å intervals (rather than the 1 Å interval in the original formula).

For each amino acid residue in a given protein structure, we first mutated the amino acid to one of 19 alternative amino acids and used the SCWRL side chain generation program [47] to rearrange the side chain of the mutated amino acid. We applied the ENCAD energy minimization protocol [48] as an intermediate step (optional in the MFS software), to minimize steric interferences. We then calculated the RAPDF values by a modified version of the potential program in the RAMP program package that uses 37 distance bins for statistical inference [32]. From the set of 20 RAPDF values for the wild type amino acid and 19 alternative amino acids, we then compiled two different summary scores.

The first summary score is the RAPDF_spread score, which is the standard deviation of the RAPDF scores for 20 mutated structures that differ in one residue, and is calculated as
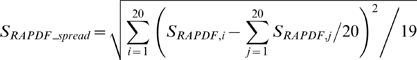



The second summary score is the RAPDF_dif score, which is calculated as
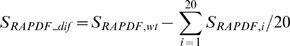
where *S_RAPDF,wt_* is the RAPDF value for the wild type structure. The RAPDF_dif score calculates the difference between wild type structure and the mean of all 20 possible structures, while the RAPDF_spread score assesses all 20 scores as a distribtion and is unrelated to the identity of the wild type amino acid. Both scores measure different aspects of structural stability induced by amino acid mutations: the RAPDF_dif score assesses the effect of the wild type amino acid on stability, while the RAPDF_spread score evaluates the potential influence of this position.

#### Amino acid type score

Since different amino acids may have different distributions in functionally important versus unimportant sites (the prior probability of an amino acid being functionally important), we also introduced a set of dummy variables into our model, representing the amino acid identity of the residue being considered. The 19 scores, *S_aatype,2_*, …, *S_aatype,20_*, are all binary variables (taking value 1 or 0) and indicate whether the corresponding amino acid is present or not (AAType).

#### Handling sequence-structure positional discordance

We used structure-based functional site datasets to benchmark the performance of our methods. Many PDB files contain chain breaks, so the use of ATOM records in sequence-based scoring schemes is unwise because the generated multiple alignments may not be accurate, especially when large chain breaks are present. In our MFS method, the two sequence-based signature scores (HMM_rel_ent, SSR) are both generated using the SEQRES records of PDB files; therefore, translation of these SEQRES-based coordinates to ATOM-based coordinates is necessary. To achieve this, we performed a global pairwise alignment of the ATOM-based sequence and the SEQRES-based sequence using the Needleman-Wunsch algorithm implemented in the EMBOSS program suite [49]. We then analyzed each aligned position to resolve the issue of SEQRES-ATOM discordances: gaps in the alignments indicate chain breaks in ATOM records, while discordant residues in alignments represent mutated residues in structure crystallization. We note that although global sequence alignments generally work well, there could be cases where very large chain breaks prevent accurate alignments; in these cases, external tools such as the S2C server (http://dunbrack.fccc.edu/Guoli/s2c/index.php) can be used in conjunction with PDB files to relate sequence to coordinates, with data obtained from XML-formatted files. The signature scores generated from the SEQRES-based sequence can then be assigned to the corresponding ATOM-based amino acid residues in the PDB file.

#### Construction of regression models

After we generated the HMM_rel_ent, SSR, RAPDF_spread, RAPDF_dif, and AAType scores, we then fit the data upon known functional sites using the following logistic regression model:
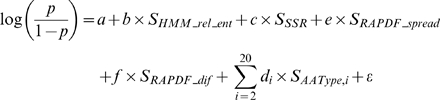
where *p* is the probability that the position is a functionally important position, *a* through *f* are model parameters, and ε is the error term. The model fitting, model checking, performance evaluation and cross validation experiments were conducted in the software STATA version 9.2 programming environment (College Station, TX).

### Performance Evaluation of the Functional Site Identification

We used the Thornton dataset [50] and the Lovell dataset [34] to evaluate the performance of MFS and its variants in identifying functional sites from protein structures. The Thornton dataset contains 1,546 enzyme active sites from 508 proteins, and the Lovell dataset contains 1,137 functional sites from 243 proteins. We evaluated the performance of functional site identification by two criteria that were used in previous studies [19]. The first criterion is the ROC score, which evaluates how the quantitative predictions on functional importance correlate with the binary assignments of whether the site is functional. This score is calculated as the area-under-the-curve by plotting the false positive rate against the true positive rate across a range of threshold values. The second criterion is the top-10 hits scores, which counts how many of the top-10 scoring residues in a given protein are also active site residues. For a given dataset, the sum of the top-10 hits scores for all proteins are used for evaluating the performance of different algorithms. In addition, we also calculated the specificity and the false positive rates for each protein, when 20% sensitivity is achieved. Assuming that TP, TN, FP, and FN represent true positive, true negative, false positive and false negative predictions, respectively, the sensitivity refers to TP/(TP+FN), precision refers to TP/(FP+TP) and the false positive rate refers to FP/(FP+TN). For the MFS and SeqonlyMFS methods, we applied five-fold cross-validation experiments to evaluate their performance: the entire dataset was divided into five parts, and during each cross validation, 80% of the proteins were used for training the model, which was then tested on the remaining 20% of the proteins.

We evaluated the performance of the MFS method by comparison to two widely used functional site identification programs for protein structures: the Evolutionary Trace server (http://mammoth.bcm.tmc.edu/report_maker) and the ConSurf server (http://consurf.tau.ac.il). We used the PDB identifier to query the Thornton and Lovell datasets using both servers with all default parameters and collected the output ZIP files from the ET server and the output “amino acid conservation score” files from the ConSurf server. Some proteins generated error messages or cannot be handled by either one of the servers and therefore were omitted from our analysis. We then used the “rho ET score” value from the ET scoring file and the conservation value from the ConSurf scoring file to evaluate the performance of these methods by the ROC and top-10 hits scores. The ET server generates many equal-valued scores (usually much more than 10) for the highest-scoring residues; therefore, the top-10 hits score was not used for ET in our comparative analysis.

For each method, we also generated modified PDB structure files in which the temperature field was replaced by the predicted functional importance scores. These structures were then visualized using the UCSF chimera software [51] so that the color of each residue represents the functional importance score value. Visual inspection of the generated structures helps to understand how and why each method worked or failed.

### Implementation of a Web Server for the Generation of MFS

We implemented the MFS generation protocol as a web server, available at http://protinfo.compbio.washington.edu/mfs. The input for this server is either a single chain sequence or structure in FASTA or PDB format, respectively, and the output is the predicted MFS score for each residue in the structure. In addition, when an input structure is provided, a new structure file with the temperature factor field replaced by the MFS scores is created to enable visual inspection of functionally important regions using molecular graphics software. If the structure file contains many chain breaks in the ATOM records, the user can additionally submit the complete sequence so that more accurate sequence alignments can be generated for the query protein. If users only submit amino acid sequence information, then the SeqonlyMFS generation protocol will be used to predict functional sites. For an average sized protein with 200 residues, the computation for SeqonlyMFS can be performed within one hour, while the computation for structure-based MFS can be performed within one day, when the processing queue is not busy. This server will be continuously updated when our MFS generation protocol is refined and improved. The standalone source code used for the MFS generation can also be downloaded at the same URL.

## Results

### Contributions of Meta-Functional Signature Components to Functional Site Identification

**Figure 1:**
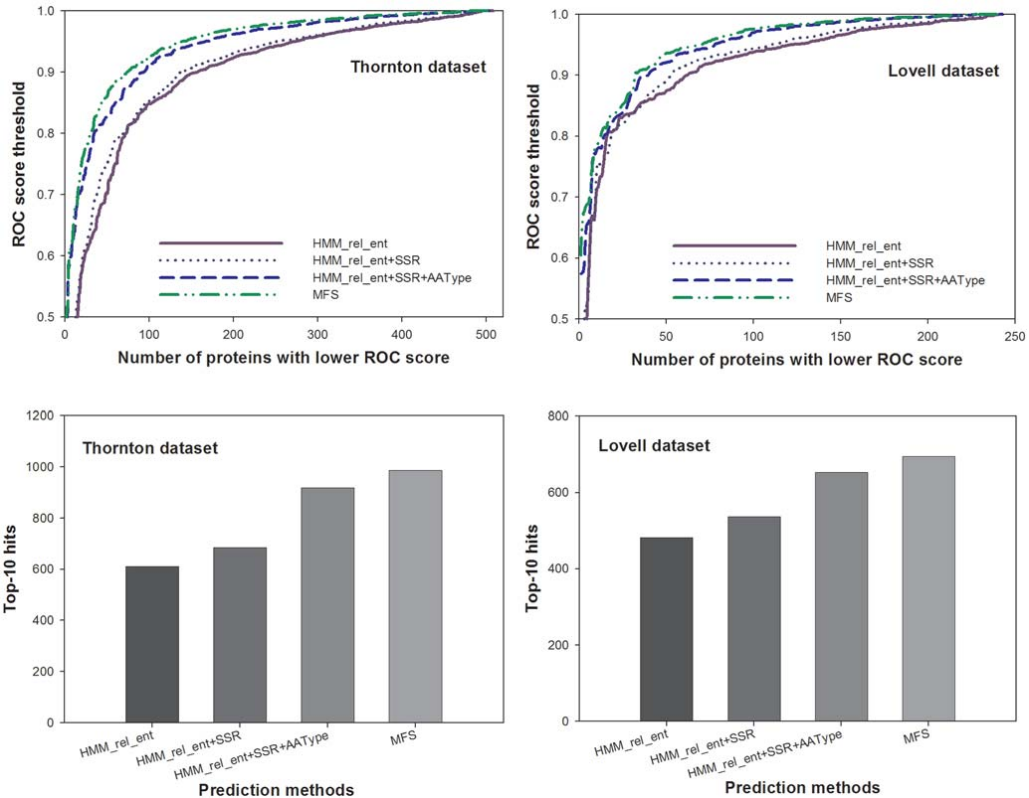
Accuracy of functional site identification in the Thornton and Lovell datasets by several methods that use sequence information only (HMM_rel_ent), then with the addition of evolutionary information (HMM_rel_ent+SSR), followed by the addition of information on the type of amino acids (HMM_rel_ent+SSR+AAType), and finally with the additional structural information (MFS). The ROC scores and the top-10 hits scores were used to evaluate performance. The four methods have increasing accuracy, demonstrating the importance of combining information from sequence, structure, evolution, and amino acid type together when functionally characterizing proteins.

Evaluating the performance of our meta-functional signature (MFS) protocols required us to use a “gold standard” functional site dataset of proteins with known structures. We did not use the “SITE” records in PDB files or “ACT_SITE” records in Swiss-Prot files because these annotations are generally not well-defined and contain high error and low coverage rates [50]. Instead, we used the Thornton dataset [50] and the Lovell dataset [34], which have been used in previous experiments [19],[36]. The Thornton dataset contains hand-annotated enzyme active sites extracted from the primary literature; the Lovell dataset contains manually compiled ligand binding sites based on literature. We used the ROC score and the top-10 hits score to evaluate performance, as previously described [19]. To investigate the added value of each component of the meta-functional signatures, we compared the performances of the incremental components of MFS: sequence conservation (HMM_rel_ent), evolutionary conservation (SSR), amino acid type (AAType), position structural stability (RAPDF_spread), and residue structural stability (RAPDF_dif) (Figure 1). Sequential incorporation of each component improves performance. The MFS using the maximum number of components has the best performance in predicting functional sites.

High correlations between components (independent variables) in a linear model will tend to destabilize the model parameters and give erroneous statistical significance. To investigate whether our MFS models have such problems, we checked the variance inflation factor (VIF). The VIF is a measure for each independent variable to estimate how collinearity among variables affects the precision of parameter estimation. VIF scores higher than 10 generally indicate problematic models. We found that all VIF scores for the parameters in MFS models when applied to both datasets are less than 4, indicating that our models do not suffer from collinearity problems. In addition, we calculated the pairwise correlation coefficients between the HMM_rel_ent score, the SSR score, the RAPDF_spread score, and the RAPDF_dif score for both datasets (Table 1). We found that the highest absolute value of correlation coefficient is 0.45 between the HMM_rel_ent and SSR scores. Therefore, each component of the MFS protocol provides additional and predominantly orthogonal information, and they can be used individually to assess the different aspects of function.

**Table 1:** Correlation coefficients of several components of the MFS method in the Thornton dataset (cells in upper-right triangle of the table) and the Lovell dataset (lower-left triangle), respectively.

	HMM_rel_ent	SSR	RAPDF_spread	RAPDF_dif
HMM_rel_ent	1.00	0.45	0.23	−0.15
SSR	0.45	1.00	0.14	−0.05
RAPDF_spread	0.23	0.16	1.00	−0.42
RAPDF_dif	−0.16	−0.06	−0.44	1.00

The components of the MFS method have a relatively low correlation with each other, demonstrating that they can provide complementary information toward accurate functional site prediction.

### Comparative Analysis of Meta-Functional Signature Performance

Several web servers have been established that assign quantitative scores to functionally important amino acid residues, and map these scores to protein structures for identifying the spatial clusters of important residues. We compared the performance of MFS with two such web servers, the Evolutionary Trace (ET) server and the ConSurf server. The ET server implements a method that combines evolutionary and entropic information to rank each residue by its functional importance [23], while the ConSurf method uses phylogenetic information to measure residue conservation [24]. Although both the ET and the ConSurf methods map the scores to protein structures, these methods do not use structural information explicitly in their calculation of functional importance. Therefore, for comparison purposes, we also used the SeqonlyMFS method, which does not use structural information.

**Figure 2:**
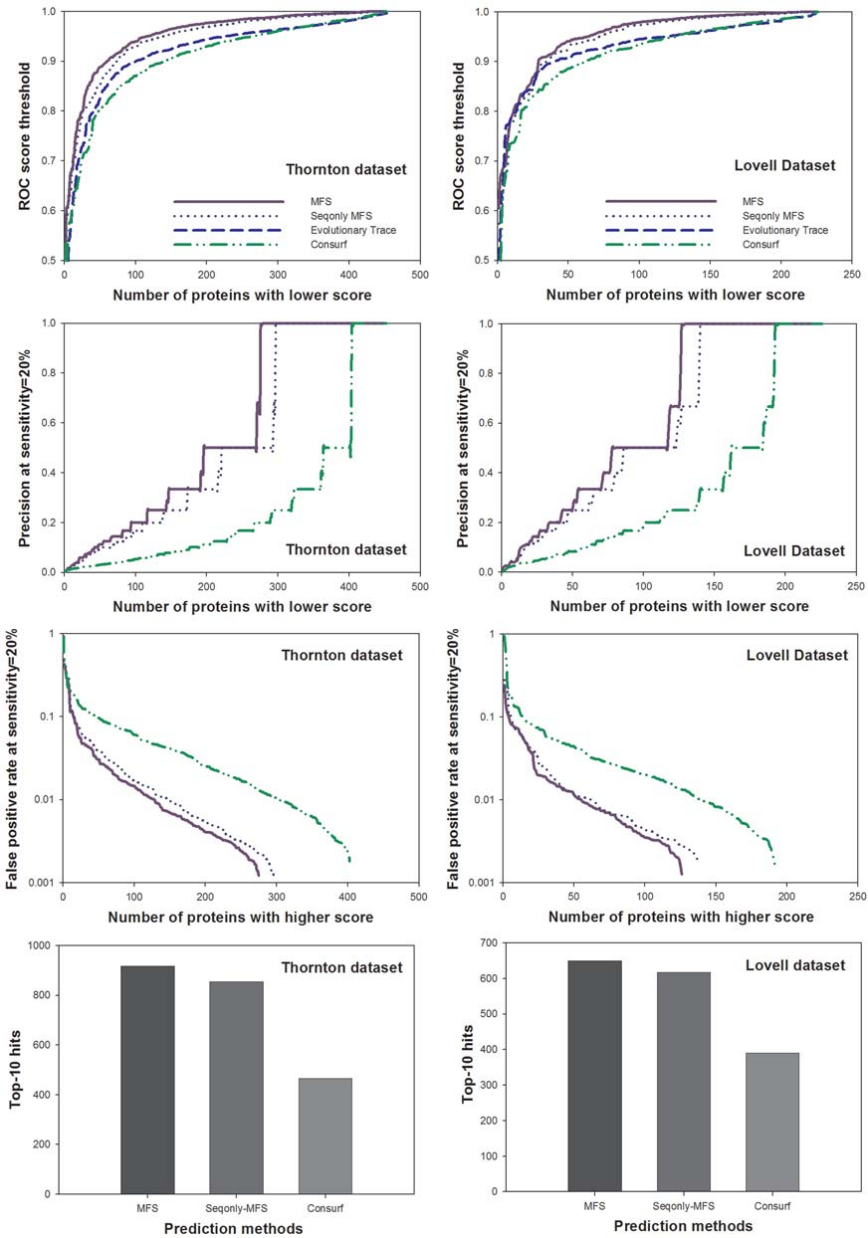
Performance comparison of the MFS method, the SeqonlyMFS method (HMM_rel_ent+SSR+AAType), the Evolutionary Trace method, and the ConSurf method with the Thornton and Lovell datasets. Only proteins for which both the Evolutionary Trace and ConSurf methods are able to give predictions are used in the comparison. Four measures are used to compare the performance, including: ROC scores, the precision when sensitivity threshold is set at 20%, the false positive rate when sensitivity threshold is set at 20% and the top-10 hits. ET is only used in the ROC score computation but not in other comparative analysis, since it gives many tied scores for top-scoring residues. Both the MFS and SeqonlyMFS methods have better performances than methods that use only one type of information.

We used the same datasets and performance measures described in the previous section to compare these methods. However, since the ET server and the ConSurf server produced error messages or could not handle some proteins, we focused our analysis on the 453/508 proteins in Thornton dataset and the 226/243 proteins in Lovell dataset for which both servers generated outputs (Figure 2). In addition, we did not calculate top-10 hits scores for the ET server, because for any given protein this server typically generates many more than 10 equal scores tied at first place. We found that MFS and SeqonlyMFS outperform both servers when their ROC measures were compared: for the SeqonlyMFS and ET comparison, the sign test P-values were 1.2e-25 and 4.4e-15 for the Thornton and Lovell datasets, respectively; for the SeqonlyMFS and ConSurf comparison, the P-values were 1.4e-39 and 1.3e-16, respectively. In addition, the SeqonlyMFS and MFS generated significantly more top-10 hits than the ConSurf server for both datasets. We note that in real-world applications, it is more important to evaluate the performance when only the most confident predictions are given; therefore, we also compared the precision measure and the false positive rate when 20% sensitivity is achieved for each protein. For both measures, MFS still has the best performance among all the methods (Figure 2). Finally, since each protein may have a variable number of functional sites, the sum of top-10 hits for all proteins may not be an optimal measure of the expected performance for a given protein. We therefore calculated the sensitivity of each method for each protein. For the Thornton dataset, the average sensitivity values for all proteins are 67.0%, 62.5%, and 33.7% for MFS, SeqonlyMFS, and ConSurf, respectively. For the Lovell dataset, the average sensitivity values are 70.0%, 66.9%, and 40.8%, respectively. Altogether, compared with methods that use only one source of information, the MFS approach that combines multiple sources of information can give improved performance in predicting functionally important residues.

### Applications of Meta-Functional Signatures

The MFS method can be regarded as a tool to define protein function as a series of quantitative values. Alternatively, when considering each component, MFS can also be treated as several vectors with equal dimensions. In previous sections we have demonstrated the application of MFS in functional site identification. Here we also demonstrate the use of MFS in other types of computational biology problems using four examples.

#### Identifying biological mechanistic residues by mapping MFS scores to protein structures

The mapping of a particular group of residues in a protein sequence to the protein structure has been proven to be a powerful way to study protein function, because human visual inspection can often reveal patterns of residue clustering and help in interpreting structure-function relationships. We applied this approach to examine how and why the MFS method works by comparing the patterns of high-scoring residue mapping generated by different methods.

#### *Ornithine decarboxylase*

**Figure 3:**
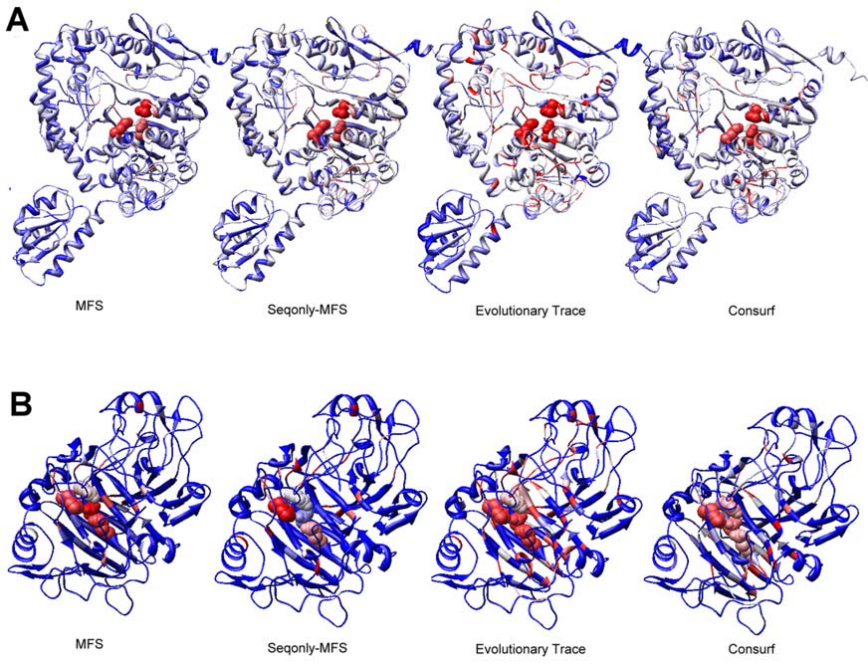
The different predictive performance of the MFS method, the SeqonlyMFS method, the Evolutionary Trace server, and the ConSurf server on two examples. The structure of an ornithine decarboxylase (A) (PDB identifier 1ord-A) and a cellobiohydrolase (B) (PDB identifier 1cel-A) are shown in the ribbon representations with the functional sites (223H-316D-355K in 1ord-A, 212E-214D-217E-228H in 1cel-A) represented as spheres. Each residue is colored by its predicted functional importance score, with the color changing from red to white to blue as the score decreases. For 1ord-A (A), both MFS and SeqonlyMFS work well in assigning the highest scores to the functional sites. However, ET and ConSurf also assign high scores to nearby residues in the surrounding cavity, thus the functional sites do not appear in the top-10 hits lists that are generated by these methods. For 1cel-A (B), all the sequence-based methods are able to assign relatively high scores to the functional sites (different shades of red color), but only the MFS method that uses structural information can boost the scores of the functional sites higher (more intense red color) to show up in the top-10 hits list.

We used the predicted functional importance scores for an ornithine decarboxylase (PDB identifier 1ord-A) as an example to illustrate the different performance of four methods: MFS, SeqonlyMFS, ET, and ConSurf. The structures are represented as ribbons, with the three functional catalytic sites (223H-316D-355K) marked as spheres, and all of the residues colored by their predicted functional importance score (Figure 3A). For this protein, 3, 2, and 0 functional sites are correctly identified in the top-10 hits by the MFS, SeqonlyMFS, and ConSurf methods, respectively (ET identifies 3 sites in its top-58 hits due to many tied scores). Therefore, detailed analysis of these structures will help us understand how and why the methods differ in their performance.

The orthinine decarboxylase has three structural domains: an N-terminal “wing”-like domain (lower left in the figure), a RLP-dependent transferase domain that contains a large cavity with a catalytic triad inside, and a small C-terminal α+β domain that partially caps the cavity (top structural domain in the figure). Both the ET and the ConSurf methods assign high scores (shown in red and light-red color) to many residues around the cavity of the protein. However, the three active sites do not gain the highest scores by these two methods, therefore the ET and ConSurf methods cannot distinguish these residues from other residues in the same cavity. In such cases, although a cluster of high-scoring residues is visually discernable, the chemically functional sites still cannot be inferred easily by these two methods. However, since both the MFS and the SeqonlyMFS methods use information based on the type of amino acids, they are able to generate higher scores for the functional sites observed in the benchmark sets (in our model, histidine, aspartic acid, and lysine have higher contributions than other types of residues), resulting in the better identification of biologically mechanistic functional sites.

#### *Cellobiohydrolase*

A second example is a cellobiohydrolase (PDB identifier: 1cel-A), which adopts a sandwich-like fold that contains multiple strands in two sheets (Figure 3B). The four functional sites (212E-214D-217E-228H) are sequentially and spatially close to each other. Only the MFS method can correctly identify 3 out of the 4 functional sites for this protein in the top-10 hits list, while the SeqonlyMFS and ConSurf methods fail to identify any (ET identifies 3 sites in its top-52 hits due to many tied scores). To make the visual inspection easier, we colored the structure so that only the relatively high scoring residues have varying shades of red and all other residues are blue. (For example, for the SeqonlyMFS method, the four functional sites are shown in white, light blue, light red and red, respectively, indicating that they have increasingly higher functional importance scores.) None of the sequence-based methods can identify the true functional sites because the sequences that correspond to this particular structural fold are highly conserved and many residues in the two sheets have relatively high conservation scores. However, since all the residues in the two beta-sheets are in close proximity to each other, the RAPDF scores are more likely to have discriminatory power to identify unfavorable residue-residue contacts, and elucidate the heavy constraints on possible amino acid substitutions. Therefore, the additional use of structural information helps the correct identification of more important residues by the MFS method.

#### Effectiveness of MFS to understand protein domains interactions

The MFS can also be used manually to gain insights into the structure and function of uncharacterized proteins, thus facilitating hypothesis generation for biochemical experiments. We have previously reported the presence of two tubulin-like genes, bacterial tubulin a (*btuba*) and bacterial tubulin b (*btubb*) in the bacteria *Prosthecobacter dejongeii* [52]. In eukaryotes, α and β tubulin form dimers and the dimers join each other to form oligomers which elongate to form protofilaments. The protofilaments constitute the microtubule cytoskeleton, which is present in all known eukaryotes but not in bacteria or archaea. Therefore, the presence of the tubulin-like genes *btuba* and *btubb* in a bacteria species caused much curiosity regarding their potential structural and functional roles as well as their evolutionary origins [52]. In our previous publication, we performed homology modeling-based structure prediction using the eukaryotic α/β-tubulin dimer as the template. We analyzed the predicted dimeric structure using RAPDF scores and concluded that *btuba* and *btubb* do not likely form dimers in bacteria due to the structural destabilizing effects of several amino acid residues in the dimer interfaces that are different between *btuba*/*btubb* and eukaryotic tubulins [52]. This finding was further supported by the fact that the electron microscopy data did not demonstrate the presence of microtubule-like structures in *Prosthecobacter dejongeii* [52]. However, in 2005, the crystal structures of *butba* and *btubb* were solved in *E.coli*, showing that *btuba* and *btubb* form dimers [53]. In addition, *in vitro* assembly analysis in *E.coli* demonstrated that *btuba* and *btubb* form protofilaments that contain equal concentrations of *btuba* and *btubb*, suggesting that the two subunits have an alternate placement along the protofilaments [54]. Therefore, we carefully re-examined why our previous predictions regarding dimer formation were wrong.

**Figure 4:**
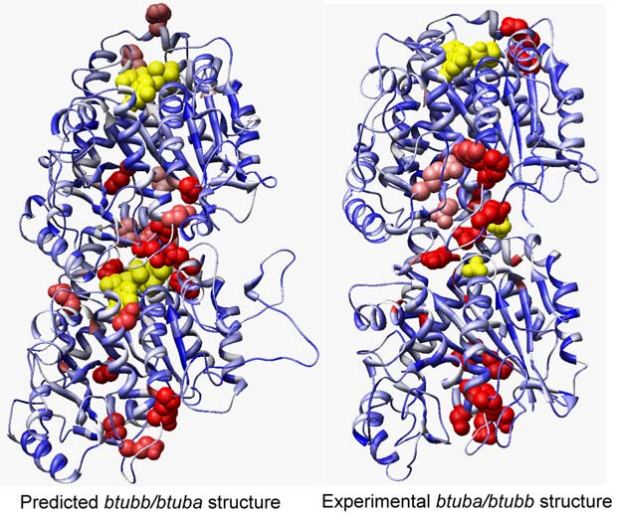
The application of MFS to understand the role of *btuba/btubb* dimer in the bacterial genus *Prosthecobacter* using the predicted and experimental structures. Both structures are colored by depicting higher MFS scoring residues with a more intense red color, with the top-10 high-scoring residues represented by spheres. One GTP and one GDP in the predicted structure, as well as one GDP and two SO_4_^2−^ ions in the experimental structure are shown as yellow spheres. The predicted structure is generated by homology-modeling techniques using the eukaryotic α/β tubulin dimer (PDB identifier: 1jff) as the template. The taxol ligand and metal ions are omitted from the predicted structure for easier depiction. In the predicted structure, *btubb* lies above *btuba*, with a GDP molecule enclosed by the dimer interface. In the experimental structure (PDB identifier: 2btq), *btuba* lies above *btubb* and there is no GDP in the dimer interface. Our MFS analysis first confirmed that *btuba* and *btubb* indeed form dimers due to the existence of a high-scoring cluster in their dimer interface, in contrast to previous predictions made by using the structural stability score alone. In addition, the MFS suggests that regardless of how *btuba* and *btubb* orient with each other, their interface is functionally important and may bind to GDP molecules.

We first compared our predicted structure in 2002 with the experimental structure that was solved in 2005 and found that the structure predictions are quite accurate: the C_α_ RMSD for *btuba* (433 residues) and *btubb* (426 residues) between predicted and experimental structures are 2.28Å and 2.36Å, respectively. We then generated the meta-functional signatures for the *btuba/btubb* dimer using the predicted structure (Figure 4, left). Our MFS generation protocol uses a slightly different structural stability score (the 37-bin RAPDF [32]) than that used in the previous publication, the 18-bin RAPDF [30]. When examining the structural stability scores of the dimer interface, we confirmed our previous predictions that dimer structures with bacteria-specific substitutions such as G100 are less stable [52]. However, when examining the top-10 residues with the highest MFS scores (20 residues depicted as red spheres in the dimer) in the entire structure, we clearly discern a cluster of high-scoring residues surrounding the GDP at the dimer interface. The MFS scores support the hypothesis that the dimer interface is indeed functionally important and binds to GDP molecules, unlike the predictions generated by structural stability alone. This example further underscores the importance of using meta-functional signatures rather than structural stability scores alone when interpreting the structural and functional roles of individual amino acid residues. In other words, although a highly accurate atomic resolution model was made, the functional sites were not accurately predicted until we evaluated the evolutionary and sequence information. Specific to this problem, we find high-scoring clusters at the head of *btuba* and the tail of *btubb*, indirectly suggesting that the tail of *btubb* may bind to the head of *btuba* in another dimer. Therefore, the MFS calculation not only supports the formation of dimers, but also the sequential addition of dimers to form protofilaments, as verified by biochemical experiments [54].

We next examined the experimental structures for the *btuba/butbb* dimer and calculated the meta-functional signatures for the dimer (Figure 4, right). Surprisingly, we found that the experimental structure for *btuba/btubb* dimer differs from our predicted structure (and also the experimental structure of the eukaryotic tubulin dimer) by the relative position of the dimer subunits. In the eukaryotic dimer, when the GDP-binding domain of α and β tubulin are oriented towards north, the α-tubulin lies above the β-tubulin so that α-tubulin binds to the GDP in the nucleotide binding domain of β-tubulin. In contrast, in the experimental structure of bacteria tubulin, *btuba* lies above *btubb*, and there is no GDP molecule in their interface, but instead there are two SO_4_^2−^ ions (shown as two small yellow spheres). Nevertheless, through MFS analysis we still found a cluster of high-scoring residues at the *btuba/butbb* interface in the experimental structures, indicating that this interface might be a functionally important binding site. Considering the relatively large gap between *btuba* and *btubb* in the dimer interface in the experimental structure, the existence of two SO_4_^2−^ ions that closely resemble the two phosphate groups in GDP, and the cluster of high-scoring residues suggested by the MFS analysis, together these pieces of evidence suggest similar interaction patterns between *btuba/btubb* in bacteria and α/β tubulin in eukaryotes despite their differences in assembly, which could be due to crystallography artifacts and/or due to the insufficient concentration of GDP molecules in solution.

Finally, by calculating the meta-functional signatures for the experimental and predicted structures for *btuba* and *btubb*, we identified a few amino acid mutations that confer the highest MFS scores for the dimeric structure. The meta-functional signatures thus suggested specific amino acids that could be introduced as mutations in the *Prosthecobacter dejongeii* tubulins for functional characterization (which would take many hours of manual analysis otherwise). Detailed biochemical and mutagenesis experiments are ongoing for these predicted important mutations. This type of detailed (and problem-specific) analysis is how we envision MFS can to be used to gain critical insights into the role of particular amino acids in protein function, and to guide experimental work.

#### Characterization of rare mechanisms in protein function using MFS

We have also applied MFS to characterize mechanisms for proteins of profoundly different function than those in the training sets, which are limited to catalytic and protein-ligand binding sites. Protein binding to biomineral surfaces is a poorly understood process. One of the few mammalian proteins known to bind the hydroxyapatite surface of bone and the only for which the mechanism has been characterized at the atomic level is osteocalcin, which thus forms an example of the applicability of MFS to predict rare mechanisms in protein function.

The osteocalcin diffraction structure (PDB identifier: 1q8h) [55] demonstrates the specific residues involved and illustrates the mechanism for the long known function of binding to the bone hydroxyapatite surface [56]. The specific placement of calcium ions along the external protein surface corresponds to the conformation of calcium ions along the exposed hydroxyapatite surface in bone [55]. As the most abundant non-collagenous protein in bone [57], osteocalcin regulates bone formation [58], and was recently shown to hold a key role in endocrine regulation of systemic metabolism [59].

**Figure 5:**
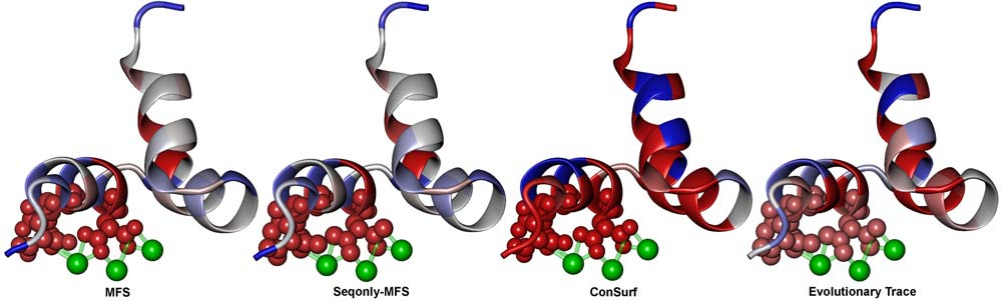
Prediction of residues with rare function not represented in the training sets. MFS was trained on a set of residues experimentally characterized to participate in canonical catalytic functionalities and protein-ligand interfaces. Protein binding to biomineral surfaces is a rare function and poorly understood process, for which the only diffraction structure available is osteocalcin binding metal ions (depicted as green spheres with ionic bonds to the γ-carboxy glutamic acid (gla) residues in transparent green tube) (PDB identifier: 1q8h). The three gla residues of osteocalcin (represented as spheres, similar to the target residues in Figure 3 above) previously shown to bind the hydroxyapatite surface of bone are clearly selected by MFS within the top six of 49 residues, with or without knowledge of structural and post-translational modification to these residues. These residues are selected within the top eight by ConSurf, with much lower discrimination from scores for the other residues in osteocalcin. None of these residues are selected within the top-10 by ET. This example demonstrates the applicability of MFS to make highly accurate and specific predictions for proteins of vastly diverse functions.

Among the top five scores, SeqonlyMFS successfully identified the three known hydroxyapatite binding residues of osteocalcin (17E-21E-24E), with the two other top five scores highlighting two cysteines involved in a fold-stabilizing disulfide bridge (23C–29C; Figure 5). The full MFS creates a similar distribution of highest functional importance, but enhances the score of a tyrosine (42Y) above two of the hydroxyapatite binding residues. Due to this high MFS score, we posit 42Y to be the phosphorylated residue (rather than the three other tyrosines) regulating the cellular signaling function for osteocalcin, which has been shown by opposing effects on pancreatic function for mice lacking osteocalcin versus those lacking the protein tyrosine phosphatase OST-PTP [59]. The slightly decreased selectivity of hydroxyapatite binding residues when including structural stability scores also corresponds to the decreased effects of mutations on stability when functional side chains are present along the free external protein surface, rather than within a compact catalytic cleft. The rigor of MFS is demonstrated here by retaining all of these residues in the top-10 scores despite the small effect on instability. Although the functional residues in the protein include three glutamic acids which are often represented as functional sites in the training datasets, we note that MFS and SeqonlyMFS provide additional information to amino acid identity information alone: first, there are five glutamic acids in the protein yet only the three true functional sites were picked out by both MFS and SeqonlyMFS; second, two Cysteins that form a disulfide bridge were correctly identified as high-scoring residues by both MFS and SeqonlyMFS, yet Cysteines are rarely represented as functional sites in our training datasets. Therefore, amino acid identity alone is not sufficient to infer functionally important residues for this protein.

In comparison, the Evolutionary Trace method fails to select the hydroxyapatite binding residues in the top-10 scores, scoring them as fourteenth through sixteenth of the thirty seven considered from the structure. Meanwhile, the ConSurf method selects these residues within the top eight scores, but provides much weaker discrimination from the rest of the protein. A large drop off in scores occurs after the first six scores in both MFS distributions, while over two thirds of residues are scored within this drop off range for ConSurf. This difference in discriminatory ability is clearly perceivable from viewing Figure 5.

The functional α-carboxy glutamic acids found by the sequence based methods in MFS were simplified in all predictions as glutamic acids, according to the coding nucleotide sequences, such that post-translational modification was not considered. This example demonstrates that MFS can be used reliably in identifying functional residues even when structure and post-translational modifications are not known, and the residues are not involved in canonical catalytic reactions or protein-ligand interactions. The identification of these modified residues indicates that MFS is directly useful in predicting sites of post-translational modification. Lastly, the structural simplification used in our analyses explains the weaker discrimination of these functional residues when considering structure, as the experimental structure is destabilized with the increased volume and negative charge of the α-carboxy glutamic acid side chains.

#### Refinement of alignments for comparative modeling

We explored the use of MFS to assist in generating pairwise alignments for distantly-related proteins. Generating accurate pairwise alignments is essential for protein structure prediction using homology modeling techniques, because generally the first step in homology modeling is to copy the atomic coordinates of the target protein to the query protein for all of the aligned residues. Some of the best alignments are produced by the 3D-Jury server [60], which is a meta-server that collects alignment information as well as scoring information from many individual servers for sequence-structure alignments and generates a consensus pairwise alignment. One of the proteins that we have worked on is the *dtx* protein with 639 residues. We submitted the query sequence to the 3D-Jury server to identify the experimental structure that has the best alignment score with the query. We used the alignment with the highest 3D-Jury score for structural modeling. One segment of the pairwise alignment that we generated is:

Query: GIREHAMGAIMNGISAFGANYKPYGGTFLNFVSYA

Target: GIAEQHAMTSAAGLAMGG–LHPVVAIYSTFLNRA

However, when we calculate the SeqonlyMFS for both the query protein and the target protein, we found that both the “H”s (histidines) in the query and the target sequence are among the top-10 high-scoring residues. This functional signature suggests that there might be an alignment error; therefore, a better alignment based on functional evidence is:

Query: GIRE-HAMGAIMNGISAFGANYKPYGGTFLNFVSYA

Target: GIAEQHAMTSAAGLAMGG—LHPVVAIYSTFLNRA

which introduces two additional gaps (generally undesirable for structure modeling) but makes the functionally important residues align with each other. Having more accurate 3D coordinates for functionally important residues and regions will be especially important in downstream function analysis and hypothesis generation for predicted structures. Since the experimental structure for this protein is not yet available, we were unable to further validate the accuracy of MFS-adjusted alignments from a structural perspective. The above procedure is merely an example of manual adjustment of pairwise alignments for distantly related proteins; however, with more sophisticated algorithmic development, it will be possible to generate functional alignments, as opposed to sequence or structure alignments, in an automated fashion for two proteins with functions that are represented by several variable length vectors. In such cases, rather than predicting functional residues, a MFS-like procedure may be used for annotation transfer between two proteins. In fact, key functional features of protein structures have already been used to improve the performance of annotation transfer between enzymes [61]. Such functional alignments would be useful for both structure prediction and functional studies of uncharacterized proteins.

## Discussion

In this work we describe a meta-functional signature (MFS) generation protocol that combines multiple sources of information for protein functional site prediction. We also demonstrate the ability of this protocol to characterize protein function on a per-residue basis using four real-world examples.

The key ideas presented in this study include the separation of structural and functional contributions, the use of pseudo-energy functions for mutated structures to determine their effects on protein function, and the combination of knowledge- and biophysics-based approaches to comprehensively annotate the functional importance of residues in a protein sequence. Most of the components of our approach are not unique: other function prediction algorithms use multiple sequence alignments, database information, and experimental and predicted protein structures. One unique aspect of our approach is in the integration of all the components into one unified knowledge- and structure-based framework that can achieve more accurate and more comprehensive predictions, yet each component can also provide different aspects of biological insight into the interpretation of protein function.

Since two different datasets (the Thornton set and the Lovell set) from different sources have been used in our study, we wish to compare and discuss the model parameters for different datasets here. This analysis may help us understand the relative contribution of the different scoring components in the two datasets. To account for the different magnitude of the predictor variables, we calculated the slope of the regression coefficient when transforming all predictors to Z-scores. For the Thornton dataset, the slope for the normalized HMM_rel_ent, SSR, RAPDF_spread, and RAPDF_dif are 1.1, 0.25, 0.52, and 0.23, respectively; for the Lovell dataset, the corresponding values are 1.1, 0.28, 0.45, and 0.19, respectively. Therefore, for the Thornton dataset that contains catalytic sites, the model contains slightly more contribution from structure-based scores, indicating that structure information is relatively more important in inferring catalytic sites than binding interfaces. In addition, we also compared the relative contribution from the 20 amino acids to the model. For the Thornton dataset, the five amino acids with the strongest contributions are Glu, Lys, Asp, Arg, and Ser, respectively, with normalized coefficients ranging from 0.55 to 0.83. For the Lovell dataset, the five amino acids with the strongest contributions are also Glu, Lys, Asp, Arg, and Ser, respectively, with normalized coefficients ranging from 0.66 to 0.84. Therefore, the amino acid identity seems to play equally important roles in these two datasets. We note that “functional residues” in the context of this study represent both catalytic sites and binding sites, yet due to the limitations of the data sources, each test dataset only contains part of the true functional sites, so some true positive hits may be mistreated as non-functional sites in each dataset. Besides comparison of two datasets, to evaluate the stability of the regression models, we have also performed similar analysis by comparing the five sets of models used in cross-validation experiments, and found that the model parameters are mostly identical between cross validations (data not shown).

Although we have presented MFS as an ensemble of scoring components integrated by a simple logistic regression model, an alternative way to integrate information is to use a sophisticated machine-learning approach, for example, via SVM based algorithms. We investigated this issue but decided to use the regression model due to several reasons: First, although SVM is well known to perform well on binary classification problems, it suffers from a lack of “biological” interpretation. For example, Petrova et al evaluated 26 different algorithms/classifiers in the WEKA software package, and presented the best combination of components as a set of seven (out of 24) residue properties for predicting catalytic residues [37]. Furthermore, Youn et al tested SVM on 314 different features, demonstrated that the combined use of multiple features improves performance, and presented the most highly ranked features [39]. Pugalenthi et al. tested 278 different features for catalytic site prediction and investigated the performance when a subset of 50–250 features are used [40]. Although these machine-learning approaches usually lead to improved performance, it is difficult to decode these “black box” methods and use an individual component (out of dozens or hundreds) to interpret different aspects of biological function, as we have done with MFS on four real-world examples. Therefore, in these cases, a simple logistic regression model is a conceptually better choice, where the regression parameters are easily intelligible. Second, functional importance may be efficiently captured by several largely independent features in a simple linear model, without resorting to testing many more complicated models and selecting the best performing model. For example, in Figure 1 of Petrova et al, although SVM ranks higher than logistic regression when comparing many different algorithms, the performance of these two methods is indeed highly similar. Therefore, we relied on a simple logistic regression model as the best approach to present and integrate an ensemble of knowledge- and biophysics-based methods in MFS.

More than just another functional site prediction algorithm, MFS can be used as a way to define protein function via a series of quantitative values that captures the functional importance of the protein. By abstracting protein function into a vector (or several vectors if each individual component is considered separately), more sophisticated algorithms can be applied to use this information more efficiently. Traditionally, two proteins can be aligned together based on their sequence similarity, structure similarity, or sequence-structure compatibility. However, the introduction of the MFS concept makes it possible to generate functional alignments between the two proteins. For example, we have demonstrated that by comparing the MFS scores for two proteins, we can potentially improve alignment accuracy using functional signatures in a manual manner. However, an automatic algorithm for aligning two variable-length matrices is non-trivial. Algorithmic advancements are needed to find an optimal solution to perform automated functional alignments for two proteins. We are actively pursuing approximate solutions to this problem.

Besides the functional site identification methods used in the paper, we realize that many other different types of methods exist to identify important residues from protein sequence or structure. Many of the methods are based on a continuous stretch of amino acid patterns, for example, the PROSITE pattern [62] and the BLOCKs pattern [63]. All residues in a given protein that match particular motifs are regarded as functionally important and the properties of the motifs may also suggest specific functional roles for the protein. However, these methods usually result in a significant over-prediction of “functional site” residues; for example, some PROSITE patterns are composed of 3-residue motifs that match multiple sites in multiple proteins. Therefore, while these methods are useful for confirming whether a pattern corresponding to a biological function exists, or for hypothesis generation to predict the possible functional category, these methods are usually too general for defining functional importance on a per-residue level. We regard our method and the motif-scanning methods as ideologically different methodologies to solve similar problems. Together they may help users gain complementary biological insights for protein characterization.

The MFS generation protocol can be enhanced in several ways. One advantage of the MFS concept is that it is composed of several independent modules, so each module can be updated and improved, without disrupting functionality of other modules. We are improving the performance of MFS from multiple aspects. First, while many other web servers (such as SIFT) use the entire NR or the entire TrEMBL sequence collection, we used only the Uniref90 data, thus allowing us to speed up BLAST searches. However, the Uniref90 dataset is not of high-quality. Many extremely short sequences exist and can be easily incorporated into the alignments and many unknown amino acids are annotated as long stretches of “X”. In addition, we used the PSI-BLAST program to scan the sequence database and generate multiple alignments, which are in fact simply the pile-up version of multiple pairwise alignments. The generation of more accurate multiple alignments will help sequence-based conservation estimations and phylogeny inferences. Furthermore, the RAPDF calculation for mutated structures can also be optimized. An optional step after side chain replacement is to minimize energy by global perturbation of the structure. This step can be implemented by the ENCAD protocol [48]. Since this procedure significantly increases execution time we made it an optional step. A faster generation of more accurate structural stability scores for mutated structures would improve MFS performance. Further development and optimization of the current protocol will greatly improve the functional annotation of sequence and structure space.

Besides improving the performance of protein functional site prediction, MFS scores treated as vectors may be used to discern functional categories for a given protein (for example, assignment of SCOP superfamily [35],[64] or a GO node in the GO hierarchy). MFS analysis also elucidates functional importance on a per-residue level, which enables the design of rational mutagenesis and biochemical experiments. Finally the MFS method may be used to modify protein function, resulting in application to protein design and drug discovery. The application of MFS protocols to many areas of computational biology and bioinformatics, as shown by examples in the paper, may significantly advance our understanding of protein sequence-structure-function relationships and guide experimental characterization of protein function.
